# MicroRNA in Pancreatic Cancer: From Biology to Therapeutic Potential

**DOI:** 10.3390/genes10100752

**Published:** 2019-09-25

**Authors:** Manmeet Rawat, Kavita Kadian, Yash Gupta, Anand Kumar, Patrick S.G. Chain, Olga Kovbasnjuk, Suneel Kumar, Gulshan Parasher

**Affiliations:** 1 Department of Internal Medicine, University of New Mexico School of Medicine, Albuquerque, NM 87131, USA; Okovbasnjuk@salud.unm.edu (O.K.); GParasher@salud.unm.edu (G.P.); 2 Department of Biotechnology, Kumaun University, Nainital, Uttarakhand 263001, India; kadian.kavita@gmail.com; 3 Department of Internal Medicine, Loyola University Medical Center, Chicago, IL 60153, USA; yashodharmangupta@gmail.com; 4 Biosecurity and Public Health Group, Bioscience Division, Los Alamos National Laboratory, Los Alamos, NM 87545, USA; akumar@lanl.gov (A.K.);; 5 Department of Biomedical Engineering, Rutgers, The State University of New Jersey, Piscataway, NJ 08854, USA; sk1350@soe.rutgers.edu

**Keywords:** microRNA, pancreatic cancer, diagnosis, prognosis, potential therapeutic targets

## Abstract

Pancreatic cancer is one of the most aggressive malignancies, accounting for more than 45,750 deaths annually in the U.S. alone. The aggressive nature and late diagnosis of pancreatic cancer, coupled with the limitations of existing chemotherapy, present the pressing need for the development of novel therapeutic strategies. Recent reports have demonstrated a critical role of microRNAs (miRNAs) in the initiation, progression, and metastasis of cancer. Furthermore, aberrant expressions of miRNAs have often been associated with the cause and consequence of pancreatic cancer, emphasizing the possible use of miRNAs in the effective management of pancreatic cancer patients. In this review, we provide a brief overview of miRNA biogenesis and its role in fundamental cellular process and miRNA studies in pancreatic cancer patients and animal models. Subsequent sections narrate the role of miRNA in, (i) cell cycle and proliferation; (ii) apoptosis; (iii) invasions and metastasis; and (iv) various cellular signaling pathways. We also describe the role of miRNA’s in pancreatic cancer; (i) diagnosis; (ii) prognosis and (iii) therapeutic intervention. Conclusion section describes the gist of review with future directions.

## 1. Introduction

Pancreatic cancer impinges profoundly on mankind and, is one of the most aggressive and fatal malignancies. Pancreatic cancer is the third leading cause of cancer-related deaths in the USA and is anticipated to take the second spot by 2020 [1,2]. In 2019, 45,750 pancreatic cancer deaths and 56,770 new cases were estimated in the USA alone [2]. Patients are often asymptomatic and thus diagnosed at a very advanced stage. Owing to the aggressive nature of this neoplasm, approximately 50% of patients already present with metastatic disease at the time of initial diagnosis [3]. Pancreas comprises of both exocrine and endocrine cells. The cancer of exocrine cells in the ducts of the pancreas which is known as pancreatic ductal adenocarcinoma (PDAC), which accounts for 90% of pancreatic cancer cases. Neoplasms arising from the endocrine cells are less common and are termed as “neuroendocrine tumors” [4]. PDAC is highly invasive and spreads to lymph nodes, liver, peritoneal cavity, lungs or the intestine, however, it rarely metastasizes to the brain or bones. In spite of a large number of on-going experimental treatment strategies for PDAC patients, the 5-year survival rate is very low. Once diagnosed and treatment has been initiated, approximately only 3–6% of patients survive up to 5 years [5,6]. The poor prognosis of PDAC patients mainly attributed to the late-stage identification and diagnosis, in combination with the invasive and aggressive nature of the neoplasm along with the resistance of pancreatic cancer cells to chemo and radiation therapies. Gemcitabine, the first-line treatment drug has moderate efficacy. Several modifications to its structures and attempts of combination therapy so far yielded only marginal improvement in gemcitabine drug efficacy [7]. Other drugs used in combination with gemcitabine such as fluorouracil, oxaliplatin, and irinotecan have yielded promising results, however, the patient survival and prognosis still remain grave [8,9]. Limitations of the existing therapeutic strategies emphasize the urgent need for improved understanding of the molecular pathways pertaining to early diagnosis, pathogenesis, and progression, in order to develop a novel more effective therapeutic regimen. 

Despite enormous progress in the field of cancer research and treatment, the biomarkers for initiation and progression of pancreatic cancer are still not well-defined. Micro RNA (miRNA) might help to establish a better diagnostic tool for pancreatic cancer. Altered and aberrant expression of miRNA is a hallmark of several human malignancies [10]. Recent reports have revealed the quintessential role of miRNA in initiation, proliferation and progression, metastasis and chemo-resistance of cancer [11]. Recently, a miRNA based therapeutic formulation has been worked out for pancreatic cancer [12].

In this review, we provide a brief account of the biogenesis of miRNA and its effects on cellular functions and miRNA related studies in pancreatic cancer patients and animal models. We also review existing literature on the role of miRNA in cell cycle and cell proliferation, apoptosis, invasion, metastasis, and cellular signaling pathways. We also explore the use of miRNA as diagnostic, prognostic and therapeutic targets in pancreatic cancer. 

## 2. Biogenesis of microRNA and Its Functions

**Figure 1:**
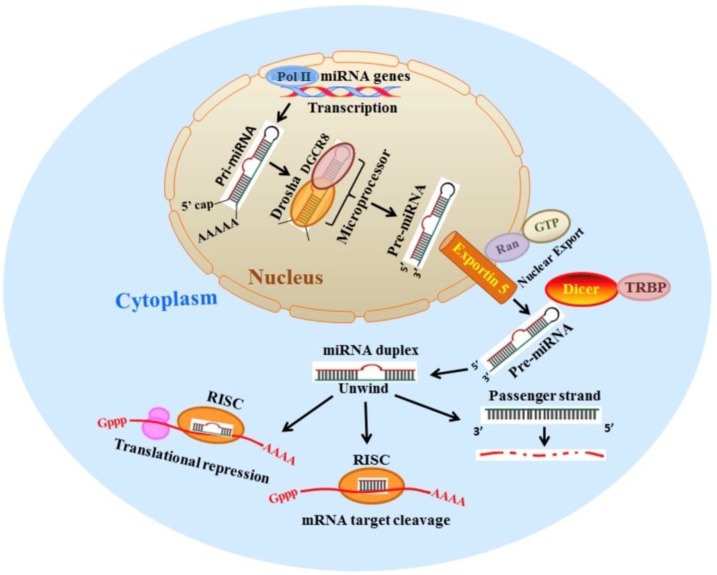
Biogenesis of miRNA and its functions. The miRNA synthesis starts in the nucleus where pri-miRNA transcript 100 to >1000 nucleotides is generated by RNA polymerase II. Subsequently, pri-miRNA is cleaved by Drosha/DGCR8 to form ~70–100 nucleotides long hairpin loop pre-miRNA. Pre-miRNA is then transported from nucleus to cytoplasm through Exportin 5 and Ran-GTP6 wherein it is further processed by RNase activity of Dicer to 19–24 nucleotides double-stranded mature miRNA duplex. The miRNA duplex then loads onto Ago in the RISC complex and undergoes strand separation. The guide strand of the miRNA mediates gene silencing by degrading the target mRNA or interfering with the translational process. The passenger strand gets degraded. Adopted from [29] © 2013 Ranganna K, Mathew OM, Milton SG, Hayes BE under a CC-BY 3.0 license.

miRNAs are 19–24 nucleotide bases of noncoding RNA that play a vital regulatory role in post-transcriptional gene expression [13,14]. miRNA was first discovered by Victor Ambros et al. in 1993 while working with *Caenorhabditis elegans* [15]. Since then, thousands of miRNAs have been identified in prokaryotic and eukaryotic organisms In humans, 2588 miRNA have been recognized with their complete biological identity, including sequence, location within the genome, and transcript annotation [16,17]. Biogenesis of miRNA requires the transcription of the miRNA gene (microRNAs can also be present in intronic regions of coding genes in eukaryotes) by RNA polymerase II to generate a longer precursor primary miRNA transcript (pri-miRNA) of variable length (100 to >1000 bps). The pri-miRNA is further processed in the nucleus by the ribonuclease complex Drosha-DGCR8 to form a hairpin-shaped intermediate pre-miRNA of 70–100 nucleotides [18,19]. To carry out the next processing step, pre-miRNA is exported to the cytoplasm with the help of transporter proteins Exportin 5 and Ran-GTP6 [20]. Inside the cytoplasm another endoribonuclease RNase III enzyme, DICER cleaves the terminal loop of the pre-miRNA to form a mature miRNA (double-stranded, 19–24 nucleotide) [21]. The mature miRNA is unwound in the cytoplasm such that one strand is degraded, and another mature strand is incorporated into the highly specialized Argonaut protein heteromultimer of the RNA induced silencing complex (RISC) [22,23]. miRNAs bind with their complementary sequence target mRNA, eventually downregulating it by either aiding cleaving of the target or by translational repression. miRNAs play a significant role in posttranscriptional gene regulation such that approximately 60% of all coding genes are estimated to be regulated by miRNA [24,25]. A single miRNA can regulate several mRNAs and vice versa, one mRNA can be a target of numerous miRNA. miRNAs play indispensable roles in several fundamental cellular processes like cell proliferation and differentiation, metabolism and apoptosis, signaling and hematopoiesis [26,27,28]. An overview of miRNA biogenesis and function is presented in Figure 1 [29].

## 3. microRNA in Pancreatic Cancer

**Figure 2:**
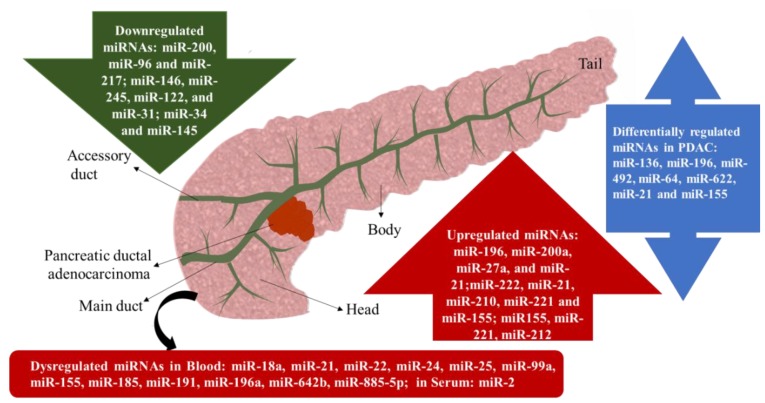
The major structures of the pancreatic gland indicating the origin of PDAC. A cataloug of commonly downregulated miRNAs (in green), upregulated miRNAs (in red) and differentially regulated miRNAs (in blue) in PDAC are listed in different colors. Differentially regulated miRNAs refers to either up or down-regulated miRNAs compared to healthy pancreatic tissue.

In the last decade, the aberrant expression of miRNA has been associated with cancer [30]. Multiple reports have demonstrated that considerable dysregulation in the expression of miRNA is a cause and consequence in numerous human tumors [31,32]. Aberrant expression of miRNA is often coupled with complete anarchy in cell regulation that includes, uncontrolled cell proliferation, tumor suppressor avoidance, cell death modulation, tumor invasion, angiogenesis, and metastasis [30,33,34]. Comparative miRNA expression profiling studies using biopsy samples from pancreas tissue of healthy individuals, PDAC and pancreatitis patients have clearly indicated the differential expression of various miRNA in cancer cells as compared to normal cells, emphasizing the potential role of miRNAs in diagnostics, prognostics and anticancer therapies [35,36]. Poy et al. conducted the first miRNA expression profiling study in mouse pancreas, followed by various other studies with a different type of samples [37]. Hong and Parking revealed that a total of 158 miRNAs were found to be differentially expressed in patient’s PDAC tissue compared to adjacent normal pancreatic tissue. A total of 51 up-regulated miRNAs that include miR-196, miR-200a, miR-27a, and miR-21 together with 107 significantly downregulated miRNAs, such as miR-200, miR-96 and miR-217 [38]. Another study conducted by Schultz et al. on formalin-fixed paraffin-embedded tissue from pancreatic ductal adenocarcinoma and normal pancreas revealed the increased expression of 43 miRNAs whereas, 41 miRNAs were found to be downregulated [39]. In patients with resectable PDAC 22 differentially expressed miRNAs were unraveled with most significant differential expression observed among gene products; miR-136, miR-196, miR-492, miR-64, and miR-622 [40]. Papaconstantinou et al. conducted differential miRNAs expression studies by employing a large number of samples from pancreatic cancer patients and normal pancreatic tissues and reported the up-regulation of miR-222, miR-21, miR-210, miR-221 and miR-155 while, the expression of miR-146, miR-245, miR-122, and miR-31 were considerably declined in pancreatic cancer tissue [41]. Consistently, other studies have also reported a clear link between the aberrant expression of miR-21 and miR-155 with the progression of cancer [42]. Irrespective of the source of samples and profiling technique used, the up-regulation of miR-155, miR-221, miR-21 and down-regulation of miR-34 and miR-145 were constantly observed among most of the miRNA differential expression studies. In pancreatic cancer patients, apart from the aberrant expression of miRNAs in pancreatic cells and tissues, miRNA dysregulation has been also observed in the systemic circulation. For example, several studies have reported enhanced levels of miR-18a, miR-21, miR-22, miR-24, miR-25, miR-99a, miR-155, miR-185, miR-191, miR-196a, miR-642b and miR-885-5p in the blood of the patients affected with pancreatic cancer [43]. miR-2 has also been observed to be upregulated in serum [44]. Like PDAC, neuroendocrine tumors of the pancreas also had aberrant expression of miRNAs. One study found upregulated miR-155, miR-146a, miR-142-5p and miR-142-3p in cancerous tissues compared with normal healthy endocrine cells of the pancreas [45]. Importantly, miRNA expression profiling studies have not only assisted in the discrimination between healthy and cancerous tissues but have also been found to distinguish between various types of pancreatic pathologies, such as acute, benign or chronic. Bloomston et al. demonstrated that the up-regulation of 21 miRNAs and downregulation of 4 miRNAs could differentiate between benign and cancerous pancreatic tissues [46]. A list of miRNA expression profile in PADC is depicted in Figure 2.

All these studies show the important role of miRNAs in various biological processes beginning from the appearance of cancerous growth until its metastasis to a distant organ. A keen insight about the specific role of miRNAs during various processes could assist in a better understanding of malignancies at a molecular level paving the way for the development of novel diagnostic and therapeutic innovations. A brief summary of differentially expressed miRNAs with their targets and implications tabulated in Table 1.

## 4. Role of miRNAs in Cell Cycle and Cell Proliferation

Cell cycle and cell proliferation are tightly regulated by various checkpoints, tumor suppressor genes, oncogenes along with other controlling mechanisms. Recent studies have uncovered the regulatory role of miRNAs in cell cycle and proliferation by modulating the expression of oncogenes and tumor suppressor genes [6,47]. The overexpression or upregulation of oncogenic miRNAs negatively affects the expression of tumor suppressor genes, resulting in the augmentation of cell proliferation. PTEN, a tumor suppressor gene is negatively regulated by the overexpression of miR-21. The product of the PTEN gene hampers the proliferation of tumor cells and also controls the numbers of cell divisions in normal cells. The overexpression of miR-21 reduces the tumor-suppressive functions of PTEN by binding to the complementary region in the 3′ UTR region of PTEN mRNA transcript [48]. miRNA also control the various cyclin-dependent kinases (CDK) and cyclin complexes that control cell cycle progression. The translation of CDK N1B gene, a CDK inhibitor is influenced by overexpression of miR-221 in pancreatic cancer [49,50]. The p27 protein controls cell cycle progression to G1 phase, by preventing the activation of either the CDK2/Cyclin E or CDK4/CyclinD complex [48,51]. miR-424-5p is another oncogenic miRNA, whose overexpression in pancreatic cancer has been reported to downregulate the SOCS6 protein and result in increased ERK pathway activity [52] that enhance cell proliferation and migration. The up-regulation of oncogenic miR-27a negatively modulates the expression of tumor suppressor Spry 2 [53].

Apart from the overexpression of oncogenic miRNAs, the stumpy expression of several tumor suppressor miRNAs has also been observed in pancreatic cancer. These miRNAs regulate the expression of proto-oncogenes. The declined expression of miR-124 in the tissues of pancreatic cancer patients affects cell cycle regulation and cell motility. miR-124 negatively regulates the expression of the Rac1 oncogene of the MKK4-JNK-C-JUN pathway and inhibits cancer cell proliferation [54]. Rac1 is a GTPase protein, with multiple functions in cell cycle regulation, cell adhesion, motility, cytoskeletal re-organizations, epithelial differentiation and protein kinase activation [52,55]. The down-regulation of miR-124 is linked with poor survival among pancreatic cancer patients.

The down-regulation of miR-203 induces cell progression to the G1 phase, resulting in enhanced cell proliferation [51]. Xu et al. recently demonstrated that the up-regulation of miR-203, significantly impedes cell proliferation, induces apoptosis, and arrests cell cycle [56]. Down-regulation of another miRNA, miR-150 in pancreatic cancer, which further downregulates the MUC4 oncoprotein as it has been shown by Srivastava et al. MUC4 down-regulation has been shown to suppress the growth and malignancy of pancreatic cancer cells [6,47].

**Figure 3:**
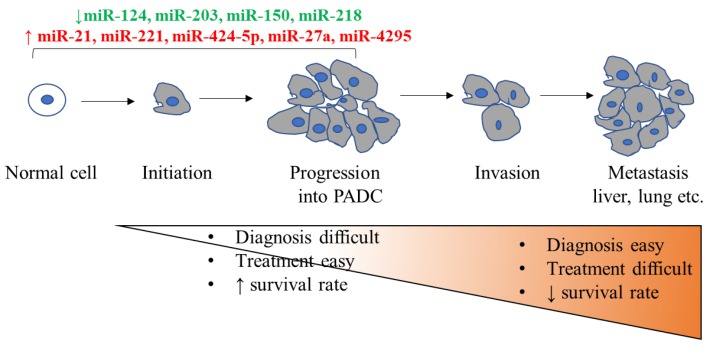
Schematic illustration of different stages of pancreatic ductal adenocarcinoma (PDAC). The list of miRNAs that are upregulated (in red color) and downregulated (in green color) during initiation and progression stages of PDAC are listed.

Along with these, there are numerous other miRNAs that regulate cell proliferation. Aberrant expression of miR-143, let-7-d, and miR-126 alter the expression of the KRAS oncogene and is correlated with abnormal cellular proliferation [51,57,58]. Cyclin E2, which is associated with the transition from G1 to S phase is directly regulated by miR-26a and is also a direct target of miR-223 [59,60]. miR-148a regulates the expression of CDC25B. The overexpression of miR-148a negatively affects the malignancy potential of pancreatic cancer cells [61]. All these studies demonstrate the important role of miRNAs in cell proliferation and growth in pancreatic cancer. A list of miRNAs that are dysregulated during initiation and progression stages of PDAC is depicted in Figure 3.

## 5. Role of miRNAs in Downregulation of Apoptosis in Pancreatic Cancer

Apoptosis or programmed cell death is a key regulatory mechanism for the maintenance of tissue homeostasis. DNA damage induces either DNA repair or apoptosis in a normal cell. However, in pancreatic cancer cells, the apoptotic pathway is likely to be perturbed, which can lead to immortality and acquisition of resistance to anticancer therapeutic agents, especially those targeting apoptotic pathways. Induction or inhibition of apoptosis is regulated by several miRNAs. miR-34a most likely regulates apoptosis by targeting Bcl-2 and Notch signaling modulators. The overexpression of miR-34a in pancreatic cancer cells, substantially downregulates the expression of Bcl-2, Notch 1 and Notch 2. The expression of miR-34a is dramatically downregulated or possibly completely abrogated in pancreatic cancer in comparison to normal tissues leading to a significant downregulation of Bsl-2, Notch 1, Notch 2 [57,62]. The overexpression of miR-155 in pancreatic cancer suppresses the pro-apoptotic gene p53 (TP53INP1). The miR-155 negatively regulates the expression of TP53INP1, resulting in the inhibition of apoptosis. Overexpression of TP53INP1 induces apoptosis and inhibits cancerous growth [63]. Aberrant expression of miR-203 also influences apoptosis. In normal cells, miR-203 regulates the expression of survivin, a baculoviral inhibitor of apoptosis. Down-expression of miR-203 in pancreatic cancer cells results in the upregulation of survivin, which in turn inhibits apoptosis [56,64]. There are several other miRNAs, which promote apoptosis such as miR-23a, miR-150, and miR-603. miR-23a regulates APAF1, which in turn induces caspase-9 with cytochrome c, which triggers apoptosis [65,66]. miR-630 and miR-150, both down-regulate the expression of IGF-1R, an insulin-like growth factor which inhibits apoptosis [67]. Recent reports also suggest that inhibition of miR-196a and overexpression of miR-24 promote apoptosis and inhibit invasion and proliferation. miR-196a along the with miR-214 targets ING4 and ING5 which in turn interacts with TP53 [68,69]. While, miR-24 directly regulates the expression of B1M which, comprise of a Bcl2 homology- 3 domains and is linked with the anti-apoptotic protein of BCL-2 superfamily [70].

## 6. Role of miRNAs in Pancreatic Cancer Invasion and Metastasis

**Figure 4:**
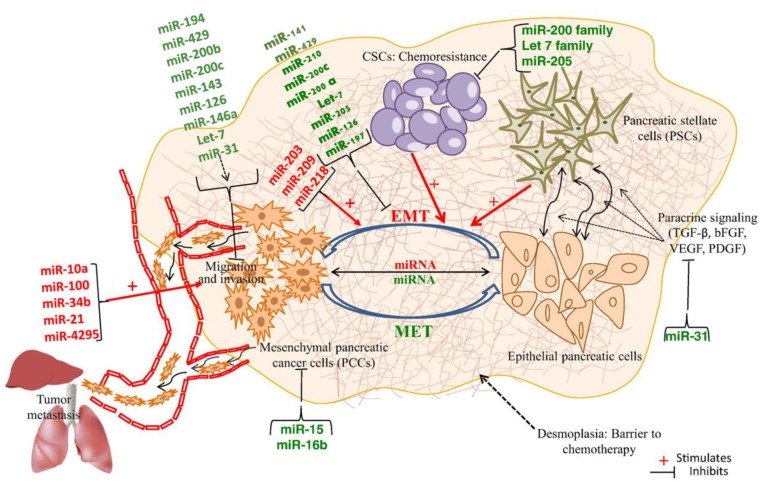
Schematic illustration of the role of tumor suppressor miRNAs at different stages of pancreatic ductal adenocarcinoma (PDAC). The figure depicts the interrelationship between pancreatic cancer cells (PCCs), pancreatic stellate cells (PSCs) and cancer stem cells (CSCs). The epithelial pancreatic cells undergo epithelial-mesenchymal transition (EMT) and become mesenchymal cells which bear high propensity to metastasize. EMT could be reversed back to mesenchymal-to-epithelial transition (MET) by the intervention of certain miRNAs. This figure is adopted and modified from [87], © 2015 Chitkara D, Mittal A, Mahato RI. under a CC BY-NC-SA 4.0 license.

Metastasis is a multi-step process and is one of the main causes of disease severity and mortality among cancer patients. It begins with the detachment of cancerous cells from the site of origin, followed by invasion into the bloodstream or lymphatic system through the basement membrane. Invasion requires epithelial to mesenchymal transition (EMT) of tumor cells, eventually, the tumor cells colonize distant sites. Thus, epithelial to mesenchymal transition (EMT) is a critical process. Various studies have reported several miRNAs regulating EMT, specifically the members of the miR-200 family (miR-141, miR-429, miR-200a, miR-200b, and miR-200c) [51,71,72,73]. Similarly, miR-203 and miR-208 can also trigger EMT [74,75]. Apart from EMT, numerous miRNAs have been identified for their role in invasion and metastasis. Up-regulation of miR-10a down-regulates homeobox transcription factors (*HOX* B1, *HOX* B2, and *HOX* A1) and induces metastasis in pancreatic cancer [76,77]. *HOX* factors are suppressors of transcription and metastasis. Huang et al. investigated the differential level of miRNA expression in metastatic and non-metastatic pancreatic cancer cell lines and observed the up-regulation of miR-100 in all metastatic cell lines [78]. Down-regulation of miR-34b has been reported in metastasis. miR-34b negatively regulates the expression of Smad 3, a promoter of metastasis [79]. Hence, low expression of miR-34b favors metastasis by up-regulating Smad 3 [79]. In 2010, Mees et al. identified the various regulatory miRNAs of a metastatic suppressor gene, EP 300 [80]. They observed a substantial up-regulation of miR-194, miR-429, miR-200b and miR-200c in metastatic adenocarcinoma cells compared to normal healthy control cells. EP 300 is negatively regulated by these miRNAs [80]. Hu et al. showed that the expression of miR-143 significantly declined in pancreatic cancer cells especially during invasion and metastasis. miR-143 negatively regulates the expression of GEF1, GEF2, K-RAS, MMP-2, and MMP-1 [57]. The down-regulation of miR-126 in PDAC results in the aberrant expression of one of its targets, ADAM9 [81]. Restoration of miR-126 has an inhibitory effect on the invasive potential of cancer cells and it reverses EMT. Like miR-126, expression of miR-146a also declines in metastatic pancreatic cancer tissue. Restoration of miR-146a significantly inhibits the invasive potential of pancreatic cancer cells by negatively regulating IRAK-1, EGFR, and MTA-2 [82]. He et al. suggested the role of miR-218 in metastatic pancreatic cancer using microarray analysis; they observed the gradual decline in the expression of miR-218 during progression from normal epithelium to pancreatic cancer to lymph node metastasis [83,84]. Up-regulation of miR-21 is correlated with liver metastasis and poor survival in the pancreatic cancer patient. Suppression of miR-21 results in the upregulation of PTEN and PDCD4, indicating that they may be the target of miR-21 [86]. Recently Yuan et al. have investigated the potential role of yet another, miR-4295, in the proliferation and invasion of pancreatic cancer cells. Further, it was suggested that a potential target of miR-4295 could be GPC5 (Glypican 5), as the suppression of miR-4295 resulted in the upregulation of GPC5, together with inhibited invasion via the stimulation of Wnt/β-catenin signaling pathway [85]. A Schematic illustration of the role of several tumor suppressor miRNAs at different stages in PDAC is highlighted in Figure 4.

## 7. Role of miRNAs in Signaling Pathways

### 7.1. KRAS Signaling Pathway

KRAS is a small membrane-bound guanosine triphosphate protein (21 kDa), belonging to the RAS family of GTPases that govern numerous signaling pathways, such as PI3K-AKT, RAF, N1-GEF, T--, MAPK and MAP2K, by altering GTP-bound (active) and GDP-bound (inactive) states in response to stimuli. Mutations in KRAS have been observed in almost 90% of PDAC patients in early stages as well as in advanced metastatic stages [88]. The mutated KRAS promotes the hyperactivation of downstream signaling pathways regulating cell survival and proliferation. Hyperactivation induces proliferation, invasion, and metastasis in pancreatic cancer. Recent studies have shown that numerous miRNAs negatively target the KRAS pathway by inhibiting the translation of, or by degrading the KRAS mRNA transcripts [89]. Here, we review the role of a few miRNAs involved in KRAS regulation in PDAC.

miR-96 binds to the KRAS at the 3′ UTR region and regulates cell proliferation. In pancreatic cancer, miR-96 is almost completely downregulated. miR-96 downregulates the expression of the HERG1 oncogene [90]. Restoration of miR-96 causes tumor suppression by negatively affecting cell proliferation, migration, and invasion [91]. Significant down-regulation has also been observed for miR-126. It also targets KRAS translation by binding to the 3′UTR of KRAS [58]. Mutated KRAS^G12D^ mediates the down-regulation of miR-143/145 via RREB (RAS- responsive element-binding protein) in pancreatic cancer cells. Mutated KRAS^G12D^ also promotes the expression of RREB1, which in response, negatively regulates the expression of miR-143/145 by binding to its promoter region. Oncogenic KRAS^G12D^, RREB, and miR-143/145 operate synchronously via a feedback mechanism [92]. A reporter assay displayed, substantial down-regulation of miR-217 in PDAC cell lines. They also revealed that restoration of miR-217 downregulates the expression of KRAS and subsequently, there is a decrease in the phosphorylation of AKT, inhibiting cell survival and proliferation [93]. Lastly, downregulation or loss of Let 7 family miRNAs in pancreatic cancer cells leads to the overexpression of KRAS, as these miRNAs negatively regulate the expression of KRAS by binding to its 3′ UTR region [94].

### 7.2. AKT Signaling Pathway

The AKT/PI3K signal transduction pathway is related to cell survival and proliferation [95]. Several miRNAs have been reported to modulate the expression of genes involved in the AKT/PI3K signaling pathways. miR-21, miR-181a, and miR-221 target PTEN, a tumor suppressor gene which is found to be mutated in many human malignancies [48,66,96,97]. PTEN controls cell proliferation by negatively regulating the PI3K-AKT pathway [98]. Up-regulation of miR-21 and miR-221 in pancreatic cancer is associated with increased cell proliferation and metastasis. Inhibition of miR-221 in pancreatic cancer cells results in the up-regulation in the expression of tumor suppressor p27, p57, PUMA and PTEN [48]. Unlike miR-181a which induces migration of pancreatic cancer cells miR-200c and miR-375 act as a suppressor of tumor growth [97]. miR-375 suppresses the malignancy in PDAC cells by targeting the mRNA transcript of PDK1, (Pyruvate dehydrogenase lipoamide kinase isozyme 1) a kinase in the AKT pathway, downstream of PI3K [99,100]. Aberrant expression of miR-200c in PDAC is correlated with MUC4 and EMT [101,102]. MUC4 has been reported to stimulate AKT, which in turn activates downstream enzymes such as N-cadherin [103,104]. 

### 7.3. JAK/STAT Signaling Pathway

The Janus kinase/signal transducer and activator of transcription (JAK/STAT) is a critical pathway that promotes cell proliferation, cell migration, differentiation, and apoptosis. The JAK/STAT pathway is stimulated by various cytokines and growth factors [105]. miR-216a negatively regulates the expression of JAK 2 and restoration of miR-216a in PDAC cells reduces tumor growth possibly by inhibition of the translation of JAK 2 downstream oncogenes, including survivin, and induces apoptosis [106,107]. Similarly, miR-130b negatively regulates the expression of STAT3 by binding to its 3′UTR region. The significant downregulation of miR-130b in PDAC cells is associated with tumor progression and poor prognosis. Restoration of miR-130b inhibits the proliferation of cancer cells [108]. In contrast, miR-155 is an oncogenic miRNA and downregulates the expression of SOCS1, a tumor suppressor of the JAK/STAT pathway. miR-155 stimulate STAT3 resulting in the invasion and migration in cancer cells [109].

### 7.4. WNT/β-Catenin Signaling Pathway

This pathway is crucial for fundamental cellular processes such as cell proliferation, fate determination, and differentiation in embryonic development. In adults, it maintains homeostasis and in cancerous cells, it plays a crucial role in proliferation, invasion, and metastasis. Binding of extracellular WNT to a receptor complex comprising of the frizzled (FZD) transmembrane receptor and low-density lipoprotein receptor-related protein 5 and 6 (LRP) [110] results in the translocation of β-Catenin into the nucleus and activation of transcription of target genes [111]. Various miRNAs target these regulators of the WNT/β-Catenin pathway. FRATL, LRP-6, FZD4, and FZD5 are upstream regulators of WNT signaling and are the direct target of miR-29c. Several studies have demonstrated the down-regulation of miR-29c in PDAC cells. Transforming growth factor-β (TGF- β) inhibits the expression of miR-29c, resulting in the aberrant, constitutive activation of WNT [112]. A microarray study demonstrated that miR-23a and miR-24 expression regulate the expression of TMEM92, HNF1-β, and FZD5 directly or indirectly [113].

### 7.5. TGF-β Signaling Pathway

The TGF-β signaling is involved in basic biological processes such as cellular growth and differentiation, and apoptosis and cellular homeostasis, in adults and developing embryo [114]. Binding of a specific ligand to the type II TGF-β receptor, recruit type I TGF-β receptors resulting in the hetero-dimerization and trans-phosphorylation and activates SMADs, In humans, there are eight distinct SMADs protein, amongst these five SMADs, are modulated by TGF-β receptor (SMAD-1, SMAD-2, SMAD-3, SMAD-5, and SMAD-8) and known as R-SMADs [115]. Activation of TGF-β/SMAD-4 signaling pathway induces the overexpression of miR-155, which in turn induces invasion and metastasis [116]. Zhang et al. have shown that the expression of SMAD 4 is directly regulated by miR-199a suggesting a regulatory role of miR-199a in TGF-β/SMAD-4 signaling [117]. Diminished expression or inactivation of SMAD-4, a tumor suppressor gene has been observed in 55% of pancreatic cancer patients. In 35% of pancreatic cancer patients, it occurs due to a deletion in both alleles whereas, in 20% of pancreatic cancer individuals, an intragenic mutation could be the reason [118].

Recent studies have shown a vital role of miRNAs in regulating the various functions of dendritic cells, which eventually affects the immune response in cancer patients. A study conducted on dendritic cells in pancreatic cancer patients showed abnormal expression of miR-146a. Aberrant expression of miR-146a is associated with the downregulation of SMAD 4, which in turn negatively affects the differentiation and antigen presentation function in dendritic cells [119]. Up-regulation of miR-421 and miR-483-3p, inhibits the expression of tumor suppressor SMAD 4, resulting in the progression of pancreatic cancer [120].

In normal healthy cells, TGF-β suppresses cancerous growth by negatively regulating C-Myc, a transcription factor. Moreover, it promotes the expression of cell cycle inhibitors including p15 and p21 [121,122]. In cancer cells, aberrant functioning of the TGF-β pathway impairs its regulatory role in cell cycle arrest. Several studies have shown a wide array of miRNAs that regulate the downstream modulators/regulators of TGF-β pathway. The maturation of miR-21 with the help of a complex comprising of DROSHA and RNA helicase p68 is induced by TGF-β signaling. The up-regulation of miR-21 promotes tumor invasion and metastasis by negatively regulating the expression of tumor suppressor genes such as PTEN and PDCD4 [123]. According to a study conducted by Gasper et al., inhibition of TGF-β receptor by small inhibitors such as SB431542, LY-2157299, and SD-208, suppressed cancer progression and metastasis in mouse models [124]. Significant down-regulation of tumor-suppressing miR-494 has been observed in PDAC cells when compared to healthy normal cells. miR-494 negatively regulates the expression of FOXM1, a transcriptional activator. Diminished expression of miR-494, boost the expression of FOXM1 and nuclear translocation of β-catenin [125]. These studies suggest a critical interplay between WNT, and SMAD proteins of the TGF-β signaling pathways with various regulatory miRNAs.

## 8. Role of miRNAs in Pancreatic Cancer Diagnosis

One of the explanations for the poor prognosis and survival in pancreatic cancer patients is the late diagnosis of the disease, as it remains asymptomatic during the early stages. Thus, there is a pressing need for the identification and development of highly specific biomarkers for early diagnosis. At present, tissues biopsies are commonly used for the diagnosis. Carbohydrate antigen 19.9 is a blood antigen, approved as a biomarker for pancreatic cancer. However, its low specificity and suboptimal sensitivity limit its diagnostic application [126]. The crucial role of miRNAs during the progression of pancreatic cancer coupled with the high stability of miRNAs at extreme pH and temperature offers great promise in the development of a miRNA-based diagnostic system [127]. Several studies have built upon this initiative by demonstrating the specific changes in the expression profile of miRNAs during different stages such as invasion, proliferation, and metastasis [46,128,129]. The advent of recent technology advances and their application, such as Next-Generation-Sequencing, customized oligonucleotide-based microarray analysis, quantitative fluorescence probe-based PCR, in situ hybridization and Northern blotting using locked nucleic acid (LNA) modified probes, have all provided a number of specific miRNA expression profiles that could be used for diagnosis [130,131,132,133,134,135,136,137]. A microarray analysis study using blood samples comparison between pancreatic cancer patients and healthy individuals clearly showed a distinct miRNA expression profile (miR-22, miR-642b, and miR-885-5p) that identifies early pancreatic cancer [138]. The overexpression of seven miRNAs that include miR-21, miR-23a, miR-31, miR-100, miR-143, miR-155, miR-2214 and down-regulation of three miRNAs i.e., miR-148a, miR-375 and miR-217 differentiate a healthy control from pancreatic cancer patients [139]. In another study, overexpression of miR-196 in tissues samples coupled with low expression of miR-217 could identify a normal pancreas from PDAC [140]. Wang et al. demonstrated, that aberrant expression of miR-21, miR-155, miR-196a and miR-210 in plasma of PDAC patients, can easily distinguish from healthy controls [141]. High level of miR-210 in the serum of pancreatic cancer patients as compared to the normal individual may be exploited as a diagnostic marker [142]. Similarly, the level of miR-192 and miR-18a in the serum and plasma of pancreatic cancer patients could identify a healthy individual from a pancreatic cancer patient [43,143]. The high concentration of miR-155 and miR-210 in the serum of pancreatic cancer patient has been reported in various studies [42,142]. Due to the increased serum concentration of miR-155 in the early stages, it could be used for the diagnosis of early pancreatic neoplasia [42]. In an extensive study, Yu et al. investigated the miRNA expression profile in PanIN lesions. They analyzed 700 miRNAs and observed the aberrant expression of 35 miRNAs. It includes the overexpression of 24 miRNAs and reduced expression of 6 miRNAs in PanIN-3 lesions. Among all these 35 miRNAs, miR-196b surfaces as a potential biomarker in identifying PanIN-3 lesions [128]. In a similar study, Li et al. evaluated 735 miRNAs in the serum of pancreatic cancer patient and analysis lead to emergences of miR-1290 as a promising biomarker [144]. miR-1290 displays higher sensitivity (81%) and specificity (80%) when compared to healthy control groups [144]. In contrast to these approaches, a combination of various biomarkers brings discernible enhancement in the sensitivity and specificity. The combination of CA 19.9 with miR-16 and miR-196a can precisely distinguish between a pancreatic cancer patient and healthy controls [145]. Similarly, when the expression profile of miR-27a-3p was coupled with CA 19.9, pancreatic cancer patient and healthy controls could be differentiated with a sensitivity of 85.3% and specificity of 81.61% [51,146]. Recently, Lu et al. have investigated the expression profile of four miRNAs (miR-7, miR-9, miR-122, and miR-14) using qRT-PCR among severe acute pancreatitis patients. They observed that the significant surge in the serum levels of these four miRNAs as compared to healthy controls [114]. 

## 9. Role of miRNAs in Prognosis

Highly specific prognostic biomarkers could assist in the clinical management of pancreatic cancer patients (Figure 4) [87]. Recent studies have shown the utility of miRNAs in predicting prognosis, especially in terms of survival. The expression profile of miR-142-5p could be used as a biomarker for gemcitabine responsiveness. Down-regulation of miR-142-5p in PDAC patient samples correlates with gemcitabine resistance, while the up-regulation is associated with enhanced patient survival [77]. Similarly, the overexpression of miR-320c is observed in gemcitabine-resistant cells [147]. In contrast, low expression of miR-200c is associated with low median survival [102]. In a comprehensive study, Greither et al. examined the connection between the upregulation of four miRNAs (miR-222, miR-203, miR-155, miR-210) and survival in a study conducted with 56 PDAC patients. An elevated level of these four miRNAs was correlated with increased mortality by 6.2 fold in comparison to low levels of these miRNAs [148]. Roldo et al. investigated the role of six miRNAs i.e., miR-105, miR-127, miR-187, miR-309-3p, miR-45 and miR-518-a-2 as prognostic biomarkers and demonstrated that the expression profile of these miRNAs may be used to predict long-term survivors among node-positive patients [30]. Several studies have revealed the importance of miR-21 as a prognostic biomarker. Analysis of samples collected from PDAC patients shows that the upregulation of miR-21 is linked with a low rate of survival in node-negative PDAC patients and metastasis to lymph nodes as well as with poor gemcitabine response in the patient [149,150,151].

## 10. Role of miRNAs as Potential Therapeutic Targets

Given the decisive role of miRNAs in various stages of pancreatic cancer, starting from its initiation to progression to metastasis; miRNAs are potential targets for the therapeutic interventions [152]. miRNA-based anticancer therapies mainly involve introduction or restoration of tumor suppressor miRNAs as “miRNA mimics” and targets an oncogenic miRNA with the help of miRNA antagonists. A miRNA mimic is a chemically modified double-stranded miRNA that is used to mimic the function of a miRNA which is downregulated or diminished due to pancreatic cancer [153]. Kent et al. demonstrated that viral-mediated transduction of tumor suppressor miRNA, miR-143/145, inhibited the tumorous growth in pancreatic cancer cells [92]. Adenovirus-mediated delivery of miR-143 also exhibited an inhibitory effect in pancreatic cancer cells by blocking the metastasis [57]. Srivastava et al. reported that the restoration of miR-150 could substantially hinder the malignant potencies and growth of pancreatic cancer cells [47]. Likewise, replenishment of miR-34 demonstrated promising results by not only inhibiting pancreatic cancer cell growth but also enhancing their sensitivity to chemo and radiation therapies [154].

A separate approach comprises of miRNA antagonists. These are single-stranded antisense oligonucleotides corresponding to target miRNA, synthesized chemically with specific modification to provide high stability, binding affinity, and protection from nucleases [155,156]. They are complementary to the guide strand of target miRNAs and inhibit its activity by binding to seed region or by interfering in the miRNA biogenesis [157,158]. Along with these antagonist miRNAs, small-molecule inhibitors are also positively employed to target miRNAs in vitro [159,160]. Targeting oncogenic miR-21 with specific small molecule antagonist have yielded promising results and shown the inhibition of growth and cell proliferation in PDAC cells [48]. Similarly, silencing of miR-10a effectively inhibits metastasis in pancreatic cancer cells and primary human tumors [76]. Repression of miR-212 and miR-132 using antagonist miRNAs also inhibited the cancerous growth [161]. Passadouro et al. have demonstrated that the co-delivery of a human serum albumin-1-palmitoyl-2-oleoyl-sn-glycerol-3-ethylphosphocholine: cholesterol with an anti-miR oligonucleotide has efficiently suppressed the up-regulation of miRNAs of pancreatic cancer (miR-10, miR-21, miR-221 and miR-222) [162,163].

## 11. Conclusions

The association of miRNAs with cancer and using these miRNAs as therapeutic targets provides new hope for the effective diagnosis and treatment of fatal pancreatic cancer. Novel data about the biogenesis, regulation, and functions of miRNAs is increasing at an exponential rate. The cardinal regulatory role of miRNAs in key biological processes such as cell survival and proliferation, invasion and metastasis, apoptosis, and drug responsiveness, highlights their potential as diagnostic, prognostic and therapeutic targets. Also, little is known about inter-cellular communication mediated by miRNAs in pancreatic cancer. Moreover, a single miRNA target several genes and a single gene may be targeted by multiple miRNAs resulting in the additive action of miRNAs in the course of the disease. The aberrant expression of miRNAs is strongly correlated with pancreatic cancer, and in at least some cases appears to play a causative role in the establishment and progression of the disease. Restoration of down-regulated tumor suppressor miRNAs and inhibition of up-regulated oncogenic miRNAs holds the enormous prospect for the development of novel effective anticancer therapies or diagnostics. While there has been tremendous progress in the field with ample ongoing progress, there remain some limitations that need to be addressed before employing miRNAs as a therapeutic target. One of the most serious limitations is the potential for off-target effects and levels of toxicity of these chemically modified miRNAs on the healthy cells of the patients. The specific delivery of therapeutics into the pancreatic cancer cells is another consideration. Nonetheless, the recently developed intravenous injections and nanoparticle associated delivery system have shown great promise. Thus far, the progress in the field of miRNAs is impressive, but there is a long way to go. Pancreatic cancer remains a leading cause of mortality but the new body of work on the function of miRNA in cancer provides hope for miRNA-based strategies for early diagnosis, improved prognosis, and better management of clinical systems.

## Figures and Tables

**Table 1:** Summary of differentially expressed microRNAs with their targets and implications in pancreatic cancer.

miRNA/s	Sample Type/Site of Action	Regulation	Target (-ve)/(+ve)	Implication	Ref.
miR-196, miR-200a, miR-27a, and miR-21	PDAC tissue/adjacent normal pancreatic tissue	Up		51↑	[38]
miR-200, miR-96, and miR-217		Down		107↓	
miR-136, miR-196, miR-492, miR-64, and miR-622	PDAC vs. healthy pancreatic duct tissue	Differential		43↑	[39]
				41↓	
miR-21 and miR-155	PDAC vs. healthy pancreatic duct tissue	Differential		Cancer progression↑	[40]
miR-222, miR-21, miR-210, miR-221 and miR-155	PDAC vs. healthy pancreatic duct large sample size	Up		43↑	[41]
miR-146, miR-245, miR-122, and miR-31		Down		41↓	[41]
miR-155, miR-221, miR-21	PDAC vs. healthy pancreatic duct tissue	Up		Common to most studies	[38,39,41,42]
miR-34 and miR-145	PDAC vs. healthy pancreatic duct tissue	Down			
miR-18a, miR-21, miR-22, miR-24, miR-25, miR-99a, miR-155, miR-185, miR-191, miR-196a, miR-642b and miR-885-5p	Blood of PDAC patients	Up		Chronic inflamation↑	[43,44]
miR-2	Serum of PDAC patients	Up			
miR-155, miR-146a, miR-142-5p and miR-142-3p	Neuroendocrine tumors/adjacent normal pancreatic tissue	Up			[45]
miR-150	Various cancer samples	Down	MUC4 (-ve)	Tumor growth↑ Malignancy↑	[6,47]
miR-21	Various cancer samples	Up	PTEN (-ve)	Oncogenic/poor survival patients	[48]
miR-221	Various cancer samples	Up	CDK N1B (-ve)	Oncogenic	[49,50]
miR-424-5p	Various cancer samples	Up	SOCS6 (-ve)	Oncogenic	[52]
miR-27a	Various cancer samples	Up	Spry 2 (-ve)	Oncogenic	[53]
miR-124	Various cancer samples	Down	Rac1 (-ve)	Oncogenic/poor survival patients	[54]
miR-143	Metastatic pancreatic cancer; microarray analysis	Down	GEF1, GEF2, K-RAS, MMP-2, and MMP-1 (-ve)	Invasive potential↑ EMT↑	[57]
miR-143, let-7-d, and miR-126	Various cancer samples		KRAS oncogene	Abnormal cellular proliferation↑	[51,57,58]
miR-26a, miR-223	Various cancer samples		Cyclin E2	Abnormal cellular proliferation↑	[59,60]
miR-148a	Various cancer samples	Up	CDC25B (-ve)	Malignancy↓	[61]
miR-34a	Various cancer samples	Down	Bcl-2, Notch 1 and Notch 2 (-ve)	Tumor growth↑ Malignancy↑	[57,62]
miR-155	Various cancer samples	Up	p53 (TP53INP1) (-ve)	Abnormal cellular proliferation↓	[63]
miR-203	Various cancer samples	Down	Survivin (-ve)	Rampant tumor growth↑ EMT↑	[64]
miR-23a	Various cancer samples	Down	APAF1	caspase-9 with cytochrome c Apoptosis↑	[65,66]
miR-630, miR-150	Various cancer samples	Up	IGF-1R (-ve)	Apoptosis↑	[67]
miR-196a, miR-214	Various cancer samples	Down	ING4 and ING5	TP53↓ Tumor growth↑	[68,69]
miR-24	Various cancer samples	Up	B1M (-ve)	Tumor growth↓ Malignancy↓	[70]
miR-200 family (miR-141, miR-429, miR-200a, miR-200b, and miR-200c)	Various metastatic cancer samples	Up/Down		Metastasis↕	[51,71,72,73]
miR-208	Metastatic cell lines	UP		Metastasis↑	[74,75]
miR-10a	Metastatic cell lines	Down	homeobox transcription factors (*HOX* B1, *HOX* B2, and *HOX* A1) (-ve)		[76,77]
miR-100	Metastatic cell lines	Up			[78]
miR-34b	Metastatic adenocarcinoma cells	Down	Smad 3 (-ve)	EMT↑	[79]
miR-194, miR-429, miR-200b and miR-200c	Metastatic adenocarcinoma cells	Up	EP 300 (-ve)	EMT↑	[80]
miR-126	PDAC progressive samples with metastasis	Down	ADAM9 (-ve)	Invasive potential↑ EMT↑	[81]
miR-146a	Metastatic pancreatic cancer; microarray analysis	Down	IRAK-1, EGFR, and MTA-2 (-ve)	Invasive potential↑	[82]
miR-218	Metastatic pancreatic cancer; microarray analysis	Down		Progression and metastasis	[83,84]
miR-4295	Pancreatic cancer cells	Down	GPC5↑	proliferation and invasion↑	[85]
